# Malignancy in anti-synthetase syndrome: clinical features and prognostic impact from a multicenter retrospective study

**DOI:** 10.3389/fmed.2026.1780337

**Published:** 2026-03-12

**Authors:** Lu Cheng, Yan Xu, Hua Wei, Yingying Gao, Hongjun He, Huaixia Hu, Yinshan Zang

**Affiliations:** 1 Department of Rheumatology and Immunology, Jiangsu Province (Suqian) Hospital, Suqian, Jiangsu, China; 2 Department of Rheumatology and Immunology, Northern Jiangsu People's Hospital, Yangzhou, Jiangsu, China; 3 Department of Rheumatology and Immunology, Nantong First People's Hospital, Nantong, Jiangsu, China; 4 Department of Rheumatology and Immunology, Taixing People's Hospital, Taixing, Jiangsu, China; 5 Department of Rheumatology and Immunology, Second People's Hospital of Lianyungang, Lianyungang, Jiangsu, China

**Keywords:** anti-Ro-52 antibody, anti-synthetase syndrome, cancer screening, interstitial lung disease, survival analysis

## Abstract

**Objective:**

To delineate the clinical characteristics, identify risk factors (including the exploratory role of anti-Ro-52 antibody), and assess the prognostic implications of malignancy in patients with anti-synthetase syndrome (ASyS).

**Methods:**

In this retrospective multicenter study, patients with idiopathic inflammatory myopathies (IIM) were analyzed, comprising 103 ASyS and 261 non-ASyS patients [including dermatomyositis (*n* = 195), immune-mediated necrotizing myopathy (*n* = 9), overlap myositis (*n* = 12), and other IIM subtypes (*n* = 45)]. Participants were stratified into four groups based on ASyS and malignancy status: ASyS with malignancy (ASyS-MAL), ASyS without malignancy, non-ASyS with malignancy (non-ASyS-MAL), and non-ASyS without malignancy. Data on demographics, clinical features, serology, and survival were collected. Multivariate logistic regression identified malignancy-associated factors, and Kaplan-Meier analysis compared survival.

**Results:**

Malignancy prevalence was 15.5% (16/103) in ASyS patients vs. 9.2% (24/261) in non-ASyS patients (*P* = 0.074). Multivariate analysis identified ASyS as an independent risk factor for malignancy (adjusted OR 2.65, 95% CI 1.15–6.11, *P* = 0.022), along with advancing age (adjusted OR 1.04 per year, 95% CI 1.01–1.07, *P* = 0.005). Comparative analysis showed ASyS-MAL patients had significantly higher creatine kinase (CK) levels (median 978 vs. 336 U/L, *P* = 0.018) and a 100% prevalence of myositis-specific antibodies (MSAs), while non-ASyS-MAL patients had a higher prevalence of heliotrope rash (75.0% vs. 37.5%, *P* = 0.019). The presence of malignancy was associated with worse overall survival (*P* < 0.001). ASyS-MAL patients had a median survival of 36.8 months, compared to 46.2 months in non-ASyS-MAL patients (log-rank *P* = 0.138).

**Conclusion:**

Anti-synthetase syndrome is an independent risk factor for malignancy in patients with myositis, challenging the traditional view of ASyS as a low-risk subtype. Systematic malignancy screening is warranted for all ASyS patients, particularly those over 60 years of age. The role of anti-Ro-52 as an independent predictor was not confirmed and requires further study.

## 1 Introduction

Idiopathic inflammatory myopathies (IIM) constitute a heterogeneous group of systemic autoimmune disorders characterized by chronic muscle inflammation and a broad spectrum of extramuscular manifestations. Among these, anti-synthetase syndrome is recognized as a distinct clinical entity, defined by the presence of autoantibodies targeting aminoacyl-tRNA synthetases (ARS), most commonly anti-Jo-1, and a classic triad of myositis, interstitial lung disease (ILD), and polyarthritis (1, 2). Recently, an international consensus has formally recommended the adoption of the acronym “ASyS” for anti-synthetase syndrome to standardize nomenclature in research and clinical practice (3). The association between IIM and an increased risk of malignancy is well established, particularly in dermatomyositis (DM) and specific autoantibody subtypes such as anti-NXP-2 and anti-TIF1γ (4–6). Historically, ASyS has been considered to carry a lower malignancy risk compared to other IIM subsets, with clinical attention predominantly directed toward its pulmonary and articular complications (7, 8).

However, this traditional perspective is increasingly being challenged by emerging reports and series documenting cases of ASyS co-occurring with various malignancies (9–12). The true prevalence, associated risk factors, and specific clinical phenotypes of ASyS patients with malignancy remain poorly characterized. This is often because of the rarity of the condition and the inherent limitations of single-center studies with small sample sizes. Furthermore, most of the existing data are derived from Western populations (13), resulting in a notable gap in large-scale, multicenter evidence on the association between ASyS and malignancy in Chinese patients. Moreover, the role of commonly co-existing autoantibodies, such as anti-Ro-52, in modulating cancer risk among the ASyS population is a topic of growing interest but remains incompletely understood (13–15).

Therefore, to address this knowledge gap regarding the link between ASyS and malignancy, this multicenter study aimed to investigate this association in a Chinese cohort and to identify specific clinical phenotypes associated with malignancy in ASyS. The primary objective of this multicenter retrospective study was to systematically assess the prevalence and identify risk factors for malignancy in a well-defined cohort of patients with ASyS. As a secondary, exploratory objective, we aimed to evaluate the specific role of anti-Ro-52 antibody in modulating cancer risk within this population. We also compared the clinical and serological features of ASyS patients with malignancy (ASyS-MAL) against those of ASyS patients without malignancy and non-ASyS myositis patients with malignancy (non-ASyS-MAL). Furthermore, we sought to rigorously evaluate the impact of malignancy on survival outcomes in this population. By addressing these objectives, this study intends to inform evidence-based malignancy screening protocols and enhance the understanding of the paraneoplastic characteristics of ASyS.

## 2 Methods

### 2.1 Study design and patient selection

This multicenter, retrospective cohort study was conducted across five tertiary academic medical centers in China. We systematically reviewed the electronic medical records (EMRs) of all consecutive patients diagnosed with IIM based on the 2017 EULAR/ACR classification criteria (with a probability score of ≥55% for “definite” or “probable” IIM) (16) who were evaluated and followed from January 1, 2012, to December 31, 2023.

The study was approved by the Institutional Review Board (IRB) under protocol number 20190048. Due to the retrospective design of the analysis, the requirement for written informed consent was waived by the respective IRBs. All patient data were anonymized and de-identified prior to analysis to ensure confidentiality and compliance with privacy regulations. Data collection was conducted by trained rheumatologists using standardized electronic case report forms (eCRFs) that were pre-piloted to ensure consistency across centers.

#### 2.1.1 Patient flow and selection

A flowchart detailing patient screening, inclusion, and exclusion is provided in Supplementary Figure S1. Briefly, a total of 410 medical records were initially screened for IIM diagnosis. After applying inclusion and exclusion criteria, 364 patients were included in the final analysis. A comparison of key baseline characteristics (age, sex, myositis, ILD) between the 46 excluded patients and the final cohort of 364 patients revealed no significant differences (Supplementary Table S1), mitigating concerns about major selection bias.

***Inclusion criteria*:** patients were included if they met all of the following criteria: fulfillment of the 2017 EULAR/ACR classification criteria for adult IIM (with a probability score of ≥55% for “definite” or “probable” IIM) (16); completion of comprehensive testing for myositis-specific antibodies (MSAs: anti-Jo-1, PL-7, PL-12, EJ, OJ, KS, Ha, Zo, Mi-2, TIF1-γ, NXP2, MDA5, SAE) and myositis-associated antibodies (MAAs: anti-Ro-52, Ro-60, PM-Scl, Ku) using a standardized commercial line immunoassay (Eurolmmun, Lübeck, Germany); and availability of at least 6 months of follow-up data from diagnosis, unless death occurred within this timeframe.

***Exclusion criteria*:** patients were excluded if they had incomplete medical records or lacked essential baseline clinical or serological data, had a prior diagnosis of other well-defined connective tissue diseases (such as systemic lupus erythematosus or systemic sclerosis) before idiopathic IIM onset, or had a confirmed malignancy diagnosed outside a standardized three-year window surrounding the IIM diagnosis date (i.e., more than 36 months before or after IIM diagnosis), to focus on the period of highest paraneoplastic risk (4, 17).

A total of 364 patients who met the inclusion criteria were included in the final analysis (Supplementary Figure S1). They were divided into two main cohorts based on anti-aminoacyl-tRNA synthetase (anti-ARS) antibody status and clinical features: the ASyS group (*n* = 103), defined by positivity for at least one anti-ARS antibody and the presence of at least one characteristic clinical manifestation (e.g., myositis, ILD, arthritis, mechanic's hands, or Raynaud's phenomenon); and the non-ASyS group (*n* = 261), consisting of patients negative for all anti-ARS antibodies; patients with classic dermatomyositis phenotype and specific antibodies strongly associated with malignancy (e.g., anti-TIF1-γ or anti-NXP2) were categorized in the non-ASyS group; and the non-ASyS group (*n* = 261), consisting of patients diagnosed with dermatomyositis (including clinically amyopathic DM; *n* = 195), immune-mediated necrotizing myopathy (IMNM; *n* = 9), overlap myositis (*n* = 12), or other IIM subtypes (*n* = 45). Each cohort was further stratified by malignancy status, resulting in four subgroups: ASyS-MAL (*n* = 16, ASyS patients with malignancy), ASyS non-MAL (*n* = 87, ASyS patients without malignancy), non-ASyS-MAL (*n* = 24, non-ASyS IIM patients with malignancy), and non-ASyS non-MAL (*n* = 237, non-ASyS IIM patients without malignancy).

Malignancy was defined as a diagnosis of invasive cancer (excluding non-melanoma skin cancer) confirmed by histopathology. For the primary analysis, only malignancies diagnosed within a standardized paraneoplastic window—from 36 months before to 36 months after the IIM diagnosis date—were considered. A sensitivity analysis using a stricter 12-month window (from 12 months before to 12 months after IIM diagnosis) was also performed to assess the robustness of the association (see Section 3.3 and Supplementary Table S2).

### 2.2 Data collection and clinical assessment

A team of trained rheumatologists collected data using a pre-piloted standardized eCRF to ensure consistency across centers, with regular meetings conducted to resolve discrepancies in data interpretation. The following variables were systematically extracted from electronic medical records:

***Demographics and clinical features***: the evaluated parameters included demographic characteristics such as age at onset of IIM, sex, and smoking history (classified as never, former, or current smoker). Myositis was assessed by the co-occurrence of proximal muscle weakness (manual muscle testing score ≤ 4/5) and elevated serum creatine kinase (CK) levels. However, because patient inclusion was based on the comprehensive 2017 EULAR/ACR criteria, the cohort also includes individuals with amyopathic or hypomyopathic presentations. These patients were correctly classified as having IIM based on their skin manifestations, serologic profiles, and imaging findings, even in the absence of significant muscle involvement. ILD was diagnosed by experienced thoracic radiologists based on characteristic findings on high-resolution computed tomography (HRCT), including ground-glass opacities, reticulations, or honeycombing. Cutaneous manifestations included heliotrope rash (violaceous erythema of the eyelids) and/or Gottron's sign or papules (erythematous to violaceous macules or papules over the metacarpophalangeal and interphalangeal joints). Additional extramuscular features comprised mechanic's hands (defined by fissuring, hyperkeratosis, and hyperpigmentation along the radial and palmar aspects of the fingers), Raynaud's phenomenon, non-erosive inflammatory arthritis, and unexplained fever (>38 °C in the absence of an identifiable infectious source).

***Laboratory parameters and malignancy characteristics***: the study assessed baseline levels of CK, lactate dehydrogenase (LDH), C-reactive protein (CRP), and erythrocyte sedimentation rate (ESR) as key laboratory parameters. Autoantibody profiles were determined using commercial line immunoassays (Euroimmun, Lübeck, Germany) and immunoblotting techniques in accordance with the manufacturer's instructions, to detect MSAs and MAAs, including anti-Ro-52, antinuclear antibodies (ANA), and rheumatoid factor (RF). All assays were performed in centralized laboratories participating in regular external quality control programs to ensure analytical consistency. Furthermore, detailed malignancy characteristics—including cancer type, histopathological diagnosis date, tumor stage, and anatomical location—were systematically recorded.

***Treatment and outcome measures*:** therapeutic approaches and survival-related parameters, including initial immunosuppressive regimens, patient survival status, and dates of last follow-up or death, were evaluated. Overall survival (OS) was defined as the time interval from diagnosis of IIM to death from any cause or the date of the last confirmed clinicalfollow-up.

### 2.3 . Statistical analyses

Statistical analyses were conducted using R software (version 4.3.0), with statistical significance defined as a two-sided *P*-value < 0.05. Continuous variables were assessed for normality using the Shapiro–Wilk test and, due to non-normal distribution, were summarized as median (interquartile range, IQR) and compared using the Mann–Whitney *U* test or Kruskal-Wallis test, as appropriate. Categorical variables were presented as frequencies (percentages) and analyzed using the Chi-square test or Fisher's exact test, depending on expected cell counts. Univariate logistic regression was performed to identify factors associated with malignancy, with variables having *P* < 0.1 retained for inclusion in multivariate analysis. Variables with *P* < 0.1 in univariate analysis were included in the multivariate logistic regression model, which was adjusted for age and sex as *a priori* confounders. Adjusted odds ratios (aORs) were estimated, and model goodness-of-fit was evaluated using the Hosmer–Lemeshow test. For survival analysis, the Kaplan-Meier method was applied, and differences in survival curves were assessed using the log-rank test. A Cox proportional hazards model, adjusted for age and sex, was used to evaluate the impact of ASyS diagnosis on survival among malignancy patients. The proportional hazards assumption was verified using Schoenfeld residuals, and individuals lost to follow-up were censored at their last known contact date.

## 3 Results

### 3.1 Patient characteristics

The baseline characteristics of the 364 included patients, stratified into four subgroups (ASyS-MAL, ASyS-non-MAL, non-ASyS-MAL, non-ASyS-non-MAL), are presented in Supplementary Table S3. The non-ASyS group (*n* = 261) was heterogeneous, comprising patients with dermatomyositis (*n* = 195), immune-mediated necrotizing myopathy (*n* = 9), overlap myositis (*n* = 12), and other IIM subtypes (*n* = 45), as detailed in the Methods. Key differences were observed across groups. ASyS subgroups (with or without malignancy) were characterized by significantly higher rates of heliotrope rash and ILD, and 100% positivity for myositis-specific antibodies (MSAs) (all *P* < 0.001). Patients with malignancy-associated IIM (both ASyS-MAL and non-ASyS-MAL) were older (*P* = 0.005 across groups) and had higher rates of myositis (*P* = 0.012).

### 3.2 . Comparative clinical phenotype: ASyS-MAL vs. non-ASyS-MAL

A detailed comparison between ASyS-MAL and non-ASyS-MAL patients is presented in Table 1. The two groups were comparable in age, sex distribution, and the prevalence of myositis, ILD, and most extramuscular features. However, significant differences were found in specific clinical and laboratory markers. Non-ASyS-MAL patients had a significantly higher prevalence of heliotrope rash (75.0% vs. 37.5%, *P* = 0.019). In contrast, ASyS-MAL patients exhibited significantly higher baseline serum CK levels (median 978 U/L vs. 336 U/L, *P* = 0.018). As per definition, all ASyS-MAL patients were positive for MSAs (100%), primarily anti-Jo-1, whereas none of the non-ASyS-MAL patients were MSA-positive (*P* < 0.001). There were no significant differences in the positivity rates for ANA, MAAs, or Anti-Ro52 antibodies between the two groups.

**Table 1:** Comparison of demographic and clinical characteristics between ASyS-MAL and non-ASyS-MAL patients.

**Variable**	**ASyS-MAL (*n* = 16)**	**Non-ASyS-MAL (*n* = 24)**	***P*-value**
**Demographics**	
Age (years, median [IQR])	61.0 [55.3–68.0]	60.0 [53.0–66.0]	0.543
Male sex	7 (43.8%)	14 (58.3%)	0.377
**Clinical features**	
Disease duration (months, median [IQR])	12 [1-72]	6 [1-12]	0.092
Fever	5 (31.3%)	3 (12.5%)	0.235
Heliotrope rash	6 (37.5%)	18 (75.0%)	**0.019** ^*^
Gottron's sign	4 (25.0%)	13 (54.2%)	0.069
Myositis	14 (87.5%)	20 (83.3%)	>0.999
Arthritis	2 (12.5%)	1 (4.2%)	0.560
Raynaud phenomenon	0 (0.0%)	0 (0.0%)	–
Mechanic's hands	2 (12.5%)	4 (16.7%)	>0.999
ILD	7 (43.8%)	7 (29.2%)	0.352
**Laboratory parameters**	
ESR (mm/1H, mean ± SD)	29.3 ± 17.1	29.8 ± 23.4	0.937
CRP (mg/L, median [IQR])	5.8 [1.3–10.0]	9.0 [1.2–40.6]	0.307
CK (U/L, median [IQR])	978 [364–3,640]	336 [99–768]	**0.018** ^*^
**Autoantibodies**	
ANA positive	6 (37.5%)	15 (62.5%)	0.131
MSAs positive	16 (100.0%)	0 (0.0%)	**< 0.001** ^*^
MAAs positive	10 (62.5%)	11 (45.8%)	0.303
Anti-Ro52 positive	10 (62.5%)	11 (45.8%)	0.303

^*^P < 0.05.

ASyS, anti-synthetase syndrome; MAL, malignancy; IQR, interquartile range; ILD, interstitial lung disease; ESR, erythrocyte sedimentation rate; CRP, C-reactive protein; CK, creatine kinase; ANA, antinuclear antibody; MSA, myositis-specific antibody; MAA, myositis-associated antibody.

Laboratory parameters (CK, ESR, CRP) represent baseline values obtained at IIM diagnosis prior to treatment initiation.

### 3.3 . Prevalence and risk factors for malignancy

The prevalence of malignancy within the paraneoplastic window in the overall IIM cohort was 11.0% (40/364). Patients with ASyS had a numerically higher malignancy prevalence compared to those without ASyS [15.5% (16/103) vs. 9.2% (24/261)], with an odds ratio of 1.82 (95% CI 0.93–3.55, *P* = 0.074).

**Figure 1:**
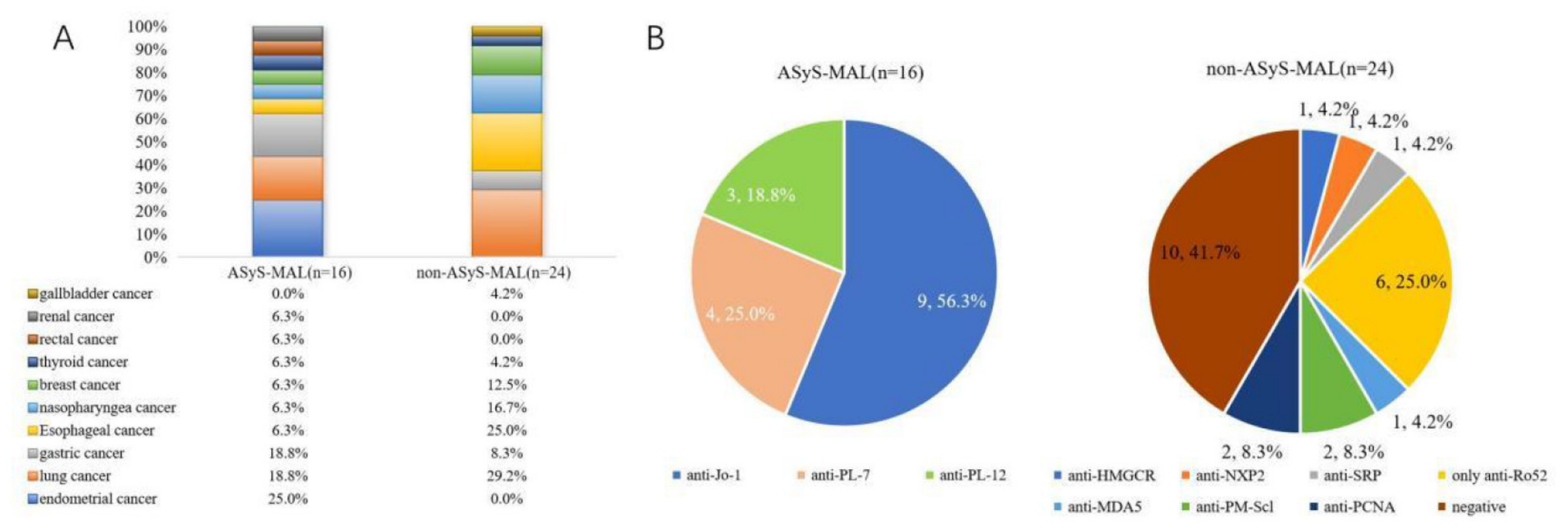
Malignancy types and autoantibody profiles in myositis patients with cancer. **(A)** Comparison of Malignancy Types between ASyS-MAL (*n* = 16) and Non-ASyS-MAL (*n* = 24) Groups. In the ASyS-MAL group, endometrial cancer (25.0%) and gastric cancer (18.8%) were the most frequent, followed by lung cancer (18.8%). Other malignancies included single cases of esophageal, nasopharyngeal, breast, thyroid, rectal, and renal cancers. In the non-ASyS-MAL group, lung cancer (29.2%) was predominant, followed by esophageal (25.0%) and nasopharyngeal (16.7%) cancers. Breast cancer accounted for 12.5%, gastric cancer for 8.3%, with single cases of gallbladder and thyroid cancer also observed. **(B)** Comparison of Myositis Autoantibody Profiles between ASyS-MAL and Non-ASyS-MAL Groups. ASyS-MAL patients exclusively exhibited anti-synthetase antibodies (100% positivity), predominantly anti-Jo-1 (56.3%). Non-ASyS-MAL patients exhibited a heterogeneous autoantibody profile, including anti-NXP2, anti-SRP, and anti-HMGCR, with 41.7% testing negative for the specific antibodies shown.

A comparison of malignancy types revealed distinct patterns between the groups. In the ASyS-MAL group (*n* = 16), endometrial cancer (25.0%, 4/16) and gastric cancer (18.8%, 3/16) were the most frequent, followed by lung cancer (18.8%, 3/16). Other malignancies included single cases of esophageal, nasopharyngeal, breast, thyroid, rectal, and renal cancers. In contrast, the non-ASyS-MAL group (*n* = 24) exhibited a predominance of lung cancer (29.2%, 7/24), followed by esophageal cancer (25.0%, 6/24) and nasopharyngeal cancer (16.7%, 4/24). Breast cancer accounted for 12.5% (3/24), gastric cancer for 8.3% (2/24), and single cases of gallbladder and thyroid cancer were also observed (Figure 1A). Antibody profiles differed significantly between the ASyS-MAL and non-ASyS myositis groups. As per definition, all ASyS-MAL patients were positive for anti-ARS antibodies, with anti-Jo-1 being the most frequent subtype (56.3%, 9/16). Other anti-ARS antibodies in this group included anti-PL-7 (25.0%, 4/16) and anti-PL-12 (18.8%, 3/16). Conversely, the non-ASyS-MAL group primarily exhibited a heterogeneous autoantibody profile. Known malignancy-associated antibodies were present in this group: anti-NXP2 was detected in 4.2% (1/24), and anti-HMGCR in 4.2% (1/24). Other autoantibodies included anti-SRP (4.2%, 1/24), and anti-MDA5 (4.2%, 1/24), and anti-PM-Scl (8.3%, 2/24), and anti-PCNA (8.3%, 2/24), and only anti-Ro52 (25.0%, 6/24). Notably, a considerable proportion (41.7%, 10/24) of patients in this group were negative for these specific autoantibodies (Figure 1B).

Univariate logistic regression analysis (Table 2) revealed that advancing age (OR 1.04 per year, 95% CI 1.02–1.06, *P* = 0.001) and the presence of anti-Ro52 antibody (OR 2.32, 95% CI 1.21–4.45, *P* = 0.011) were significantly associated with an increased risk of malignancy. ASyS diagnosis showed a trend toward significance (OR 1.85, 95% CI 0.96–3.58, *P* = 0.067). In the multivariate model adjusted for age and sex, both ASyS diagnosis (aOR 2.65, 95% CI 1.15–6.11, *P* = 0.022) and increasing age (aOR 1.04 per year, 95% CI 1.01–1.07, *P* = 0.005) remained significant independent risk factors for malignancy. The transition from a trend in the univariate analysis (*P* = 0.067) to a statistically significant independent association in the multivariate model is likely explained by the adjustment for age, a potent confounder. Diagnostic tests supported model robustness: the Hosmer-Lemeshow goodness-of-fit test was non-significant (*P* = 0.412), and variance inflation factors for all covariates were below 2, indicating no substantial multicollinearity. Sensitivity analysis using a 12-month paraneoplastic window yielded consistent results (aOR for ASyS: 2.41, 95% CI 1.08–5.39, *P* = 0.032; Supplementary Table S2).

**Table 2:** Univariate and multivariate analysis of risk factors for malignancy in myositis patients.

**Risk factor**	**Univariate analysis**		**Multivariate analysis**	
	**OR (95% CI)**	***P*** **-value**	**aOR (95% CI)**	***P*** **-value**
Age (per 1-year increase)	1.04 (1.02–1.06)	0.001^*^	1.04 (1.01–1.07)	0.005^*^
ASyS diagnosis (vs. non-ASyS)	1.85 (0.96–3.58)	0.067	2.65 (1.15–6.11)	0.022^*^
Anti-Ro-52 positive	2.32 (1.21–4.45)	0.011^*^	2.08 (0.92–4.71)	0.079
ILD presence	0.72 (0.38–1.38)	0.320	–	–
CRP (per 10 mg/L increase)	1.06 (0.97–1.16)	0.188	–	–

^*^P < 0.05. OR, odds ratio; aOR, adjusted odds ratio; CI, confidence interval.

### 3.4 . Survival and prognosis

**Figure 2:**
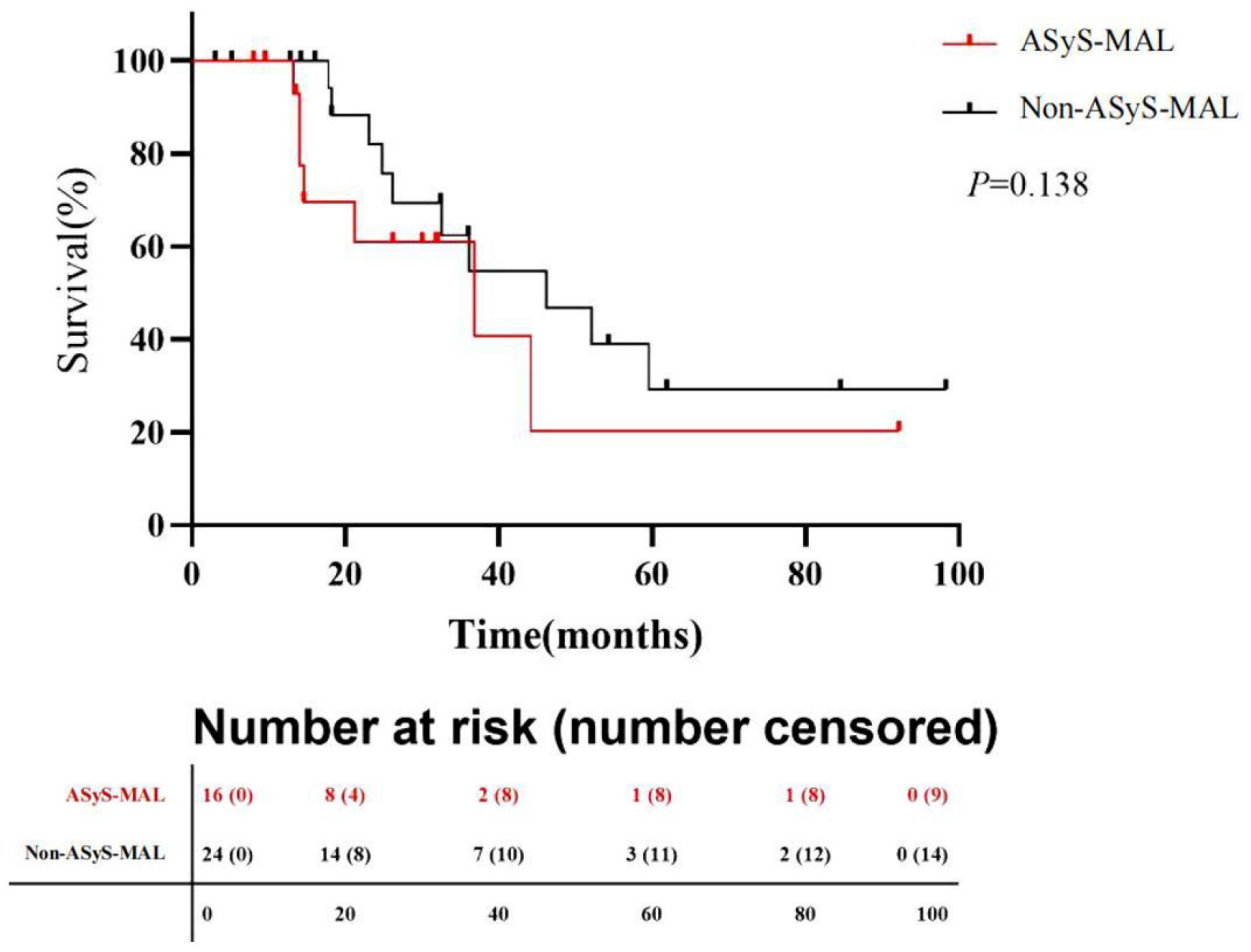
Overall survival of myositis patients with malignancy, stratified by ASyS status. Median survival was 36.8 months in the ASyS-MAL group compared to 46.2 months in the non-ASyS-MAL group. Among cancer patients, those with ASyS-MAL may experience a poorer prognosis compared to Non-ASyS-MAL patients; however, the survival difference between the two groups did not reach statistical significance in this cohort (log-rank *P* = 0.138).

With a median follow-up of 30.4 months, 23 deaths occurred in the overall malignancy cohort (*n* = 40). The causes of death were malignancy progression (*n* = 16), respiratory failure due to ILD (*n* = 4), infection (*n* = 2), and other causes (*n* = 1). Survival analysis demonstrated that patients with ASyS-MAL had a significantly poorer prognosis compared to those without ASyS-MAL. From the time of myositis diagnosis, the median survival was 36.8 months in the ASyS-MAL group, whereas it was 46.2 months in the non-ASyS-MAL group. However, the difference in survival distribution between these two subgroups did not reach statistical significance in this cohort (log-rank *P* = 0.138), likely due to the limited sample size (Figure 2).

## 4 Discussion

This multicenter study provides evidence that anti-synthetase syndrome is an independent risk factor for malignancy in patients with idiopathic inflammatory myopathies. While the observed malignancy prevalence was numerically higher in the ASyS cohort compared to the non-ASyS cohort, this difference did not reach statistical significance in the overall cohort analysis, likely constrained by sample size. Nevertheless, its persistence as a significant factor in the multivariate model underscores a clinically meaningful association that challenges the traditional view of ASyS conferring a lower neoplastic risk.

Contrary to our initial hypotheses, the comparative analysis between ASyS-MAL and non-ASyS-MAL patients revealed no significant differences in age or the prevalence of interstitial lung disease. However, a distinct phenotypic profile emerged for ASyS-MAL: these patients exhibited significantly higher CK levels and, by definition, a 100% prevalence of MSAs. Elevated CK may reflect a state of more active muscle inflammation, potentially driven by specific antibody subtypes such as anti-Jo-1, which are known to be associated with prominent myopathic injury (11). Conversely, non-ASyS-MAL patients presented with a significantly higher frequency of heliotrope rash, aligning more closely with the classic cutaneous phenotype of dermatomyositis. These distinctions highlight the importance of integrating clinical features with antibody profiles for subtype characterization.

The heterogeneity of autoantibodies in the non-ASyS-MAL group warrants further discussion. This group included patients with well-established malignancy-associated antibodies, such as anti-NXP2, which are known to confer a high risk of cancer in dermatomyositis (6). The presence of these high-risk antibodies in the comparator group makes our finding that ASyS remained an independent risk factor even more compelling. After adjusting for age and sex, the diagnosis of ASyS itself carried a 2.65-fold increased odds of malignancy, suggesting that the risk associated with ASyS is not merely a reflection of these other high-risk antibody specificities.

A sensitivity analysis comparing ASyS patients directly to the DM subgroup (*n* = 195) further supports this finding. In this analysis, ASyS remained significantly associated with increased malignancy risk after adjustment (aOR 2.41, 95% CI 1.05–5.55, *P* = 0.039; Supplementary Table S4). This indicates that even when compared to a group with a well-characterized elevated cancer risk (DM), ASyS confers an additional, independent risk.

Regarding anti-Ro-52, its strong univariate association with malignancy was attenuated in the multivariate model. This suggests that its risk signal is largely collinear with the diagnosis of ASyS itself, rather than an independent predictor (18, 19). In our cohort, anti-Ro-52 was highly prevalent in the ASyS groups (62.5% in ASyS-MAL, 79.3% in ASyS-non-MAL), confirming its role as a common companion antibody in ASyS rather than a primary driver of cancer risk. This collinearity explains its loss of statistical significance in the adjusted model. It is noteworthy, however, that anti-Ro-52 is frequently present in ASyS and has been linked to greater disease severity and activation of the interferon pathway (20, 21), warranting continued clinical vigilance in seropositive patients.

Furthermore, the distribution of malignancy types in our cohort revealed a distinctive pattern, with endometrial and gastric cancers collectively representing 43.8% of cancers in the ASyS-MAL group. This contrasts with the non-ASyS-MAL group, where lung cancer (29.2%) was most prevalent, followed by esophageal (25.0%) and nasopharyngeal (16.7%) cancers. The relatively high frequency of gastric cancer (18.8%) among ASyS-MAL patients prompts consideration of organ-specific immune interactions. Dysregulation within the tumor microenvironment, particularly involving immune checkpoint molecules, may provide a link. Soluble Human Leukocyte Antigen-G (sHLA-G) is a pivotal immunoregulatory molecule that inhibits NK cell and T-cell function, thereby facilitating tumor immune escape (22). Critically, HLA-G expression has been directly documented in gastric carcinoma tissue, where its presence is associated with specific clinicopathological features and may impact prognosis (23). Although our study did not measure sHLA-G levels, it is plausible that the systemic inflammatory and autoimmune milieu characteristic of ASyS could predispose to an upregulation of such immunomodulatory molecules in susceptible tissues. This could create a permissive local environment for the development or progression of specific cancers, such as gastric cancer. Future studies directly assessing sHLA-G in ASyS patients, stratified by malignancy status, are warranted to explore this hypothesis.

Although not statistically significant, the observed trend toward shorter median survival in ASyS-MAL patients (36.8 months vs. 46.2 months) may reflect the compounded burden of managing both progressive ILD and malignancy. However, this finding must be interpreted with caution due to the limited sample size and lack of statistical significance. The concurrence of progressive, often treatment-refractory ILD and an aggressive malignancy creates a “dual burden” that complicates therapeutic management. Immunosuppressive regimens essential for controlling ASyS may inadvertently facilitate tumor progression, while certain chemotherapeutic agents carry the risk of exacerbating underlying ILD (24). Consequently, the management of ASyS-MAL necessitates a multidisciplinary framework integrating rheumatology, oncology, and pulmonology expertise to navigate these complex trade-offs.

Our study has several limitations. Its retrospective design carries inherent risks of selection and information bias. The small sample size of the ASyS-MAL subgroup limited the statistical power for several comparisons, including the survival analysis and likely contributed to the borderline significance of anti-Ro-52 in the adjusted model. The non-ASyS group was heterogeneous, comprising various IIM subtypes with differing inherent cancer risks. This heterogeneity, while addressed in part by a sensitivity analysis (Supplementary Table S4), may affect the precision of the comparative risk estimate. A direct comparison of ASyS with specific high-risk antibody subgroups (e.g., anti-TIF1-γ) was not feasible and should be explored in future collaborative studies. Data on potential confounders such as detailed smoking history or family cancer history were not uniformly available. The absence of uniform, detailed data on important cancer risk factors such as smoking pack-years and family history of malignancy represents a significant limitation. These unmeasured confounders could not be adjusted for in our multivariate model and may influence the observed associations. Despite these limitations, the strengths of this study include its multicenter design, well-characterized patient population, and robust statistical methodology.

In conclusion, our findings position anti-synthetase syndrome as an independent risk factor for malignancy in myositis patients. We recommend a heightened index of suspicion and systematic malignancy screening for all ASyS patients, with particular emphasis on those over 60 years of age. While anti-Ro-52 antibody positivity was common in our ASyS-MAL cohort, its lack of independent significance in our adjusted model suggests it should be considered in the overall clinical context rather than as a primary screening criterion. Future large-scale prospective studies are essential to refine optimal screening strategies and to elucidate the complex biological mechanisms linking antisynthetase autoimmunity to oncogenesis.

## Data Availability

The raw data supporting the conclusions of this article will be made available by the authors, without undue reservation.
