# Supplementary figures and images for: Malignancy in anti-synthetase syndrome: clinical features and prognostic impact from a multicenter retrospective study
Source: Front Med (Lausanne). 2026 Mar 12;13:1780337. doi: 10.3389/fmed.2026.1780337 (PMC13018139; doi:10.3389/fmed.2026.1780337)

# Supplementary Figure S1

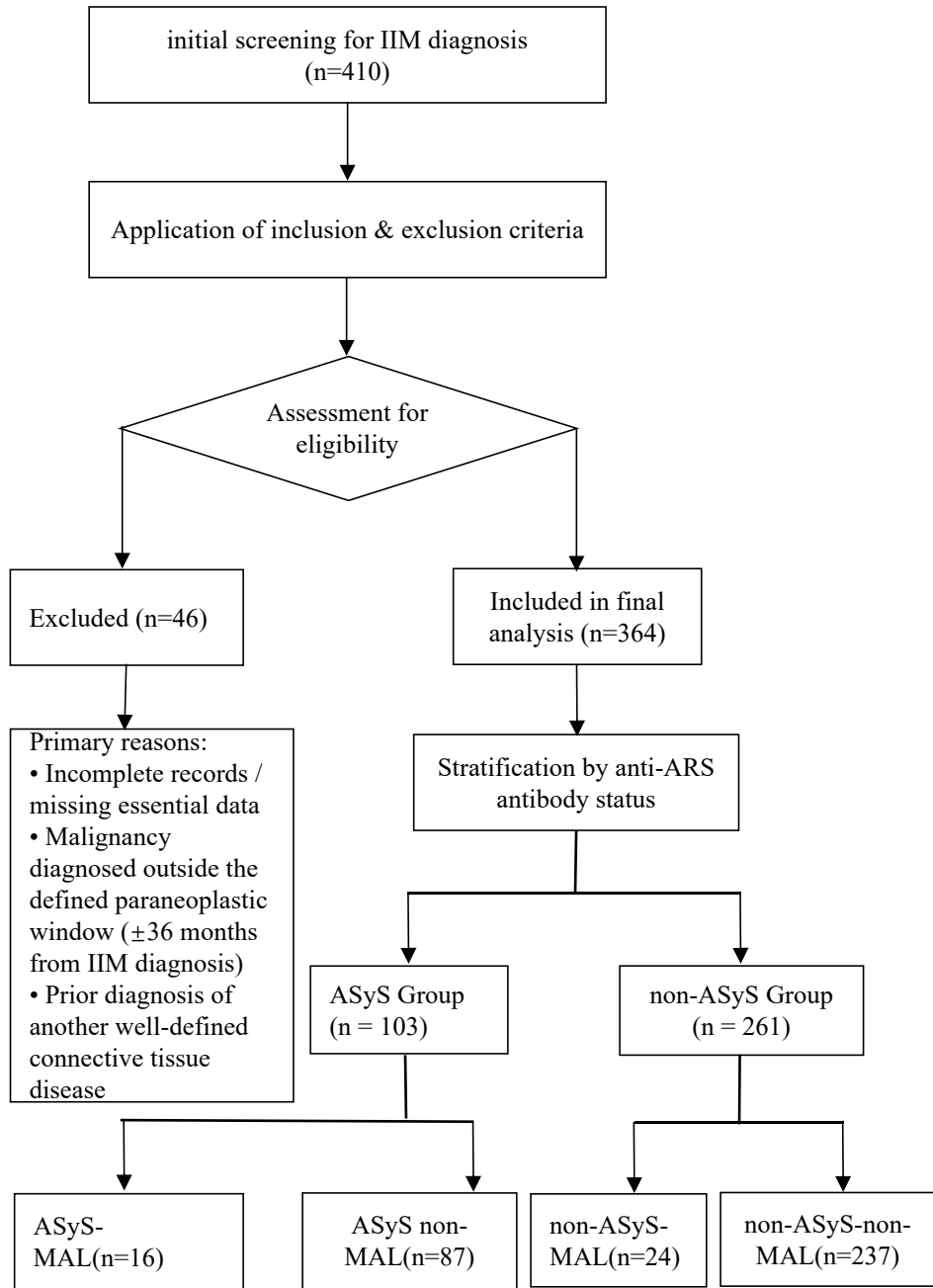

Supplement: Supplementary file 1 [file Image_1.pdf]
