# Supplementary material for: Malignancy in anti-synthetase syndrome: clinical features and prognostic impact from a multicenter retrospective study
Source: Front Med (Lausanne). 2026 Mar 12;13:1780337. doi: 10.3389/fmed.2026.1780337 (PMC13018139; doi:10.3389/fmed.2026.1780337)
Supplement: Supplementary file 2 [file Table_1.pdf]

Supplementary Table S1. Comparison of Baseline Characteristics Between Excluded and Included Patients

Characteristic	Excluded Patients (n=46)	Included Patients (n=364)	P-value
**Age at IIM onset (years), median (IQR)**	54.6 (49.0–66.0)	54.0 (45.0–64.0)	0.764
**Male sex, n (%)**	17 (37.0%)	126 (34.6%)	0.753
**Myositis, n (%)**	38 (82.6%)	277 (76.1%)	0.324
**Interstitial lung disease, n (%)**	26 (56.5%)	220 (60.4%)	0.609
**Anti-ARS antibody positive, n (%)**	9 (19.6%)	103 (28.3%)	0.210
**Malignancy, n (%)**	6 (13.0%)	40 (11.0%)	0.677

Abbreviations: IQR, interquartile range; ARS, aminoacyl-tRNA synthetase.

Note: Patients were excluded primarily due to incomplete medical records (n=28), diagnosis of malignancy outside the paraneoplastic window (n=12), or prior diagnosis of other connective tissue diseases (n=6). No significant differences were observed in key baseline characteristics between excluded and included patients, suggesting minimal selection bias.
