# Supplementary material for: Malignancy in anti-synthetase syndrome: clinical features and prognostic impact from a multicenter retrospective study
Source: Front Med (Lausanne). 2026 Mar 12;13:1780337. doi: 10.3389/fmed.2026.1780337 (PMC13018139; doi:10.3389/fmed.2026.1780337)
Supplement: Supplementary file 3 [file Table_2.pdf]

Supplementary Table S2. Sensitivity Analysis of Malignancy Risk Using a 12-Month vs. 36-Month Paraneoplastic Window

Risk Factor	12-Month Window	36-Month Window	Comparison
**ASyS Diagnosis**			
aOR (95% CI)	2.41 (1.08–5.39)	2.65 (1.15–6.11)	Similar magnitude and significance
P-value	0.032*	0.022*	
**Age (per year)**			
aOR (95% CI)	1.04 (1.01–1.07)	1.04 (1.01–1.07)	Identical
P-value	0.005*	0.005*	
**Anti-Ro52 Positive**			
aOR (95% CI)	1.95 (0.85–4.49)	2.08 (0.92–4.71)	Slightly attenuated
P-value	0.112	0.079	
**Number of Malignancy Cases**	28/364 (7.7%)	40/364 (11.0%)	30% reduction in cases

Interpretation: The independent association between ASyS and malignancy remained significant (aOR 2.41, *P*=0.032) even when applying a stricter 12-month paraneoplastic window, supporting the robustness of our primary finding. The effect size was similar to that observed with the 36-month window (aOR 2.65).
