# Supplementary material for: Malignancy in anti-synthetase syndrome: clinical features and prognostic impact from a multicenter retrospective study
Source: Front Med (Lausanne). 2026 Mar 12;13:1780337. doi: 10.3389/fmed.2026.1780337 (PMC13018139; doi:10.3389/fmed.2026.1780337)
Supplement: Supplementary file 4 [file Table_3.pdf]

Supplementary Table S3: Comparison of Baseline Characteristics Among Four IIM Subgroups

Variable	ASyS-MAL (n=16)	ASyS-non-MAL (n=87)	non-ASyS-MAL (n=24)	non-ASyS-non-MAL (n=237)	*P*-value
**Demographics**					
Age (years, median [IQR])	61.0 [55.3-68.0]	55.0 [46.0-63.0]	60.0 [53.0-66.0]	52.0 [42.0-63.0]	**0.005***
Male sex	7 (43.8%)	27 (31.0%)	14 (58.3%)	78 (32.9%)	0.098
Smoking history	3(18.8%)	21(24.1%)	9(36.8%)	35(25.5%)	0.456
**Clinical Features**					
Disease duration (months, median[IQR])	12[2-72]	12[2-24]	6[1-12]	12[2-36]	0.678
Fever	5 (31.3%)	29 (33.3%)	3 (12.5%)	63 (26.6%)	0.234
Heliotrope rash	6 (37.5%)	10 (11.5%)	18 (75.0%)	114 (48.1%)	<0.001*
Gottron's sign	4 (25.0%)	16 (18.4%)	13 (54.2%)	112 (47.3%)	<0.001*
Myositis	14 (87.5%)	62 (71.3%)	20 (83.3%)	175 (73.8%)	0.012*
Muscle strength (0-5 grade, mean±SD)	3.9±1.5	4.1±0.9	4.3±0.8	4.2±1.1	0.789
Arthritis	2 (12.5%)	23 (26.4%)	1 (4.2%)	34 (14.3%)	0.654
Raynaud phenomenon	0 (0.0%)	10 (11.5%)	0 (0.0%)	19 (8.0%)	0.245
Mechanic's hands	2 (12.5%)	17 (19.5%)	4 (16.7%)	50 (21.1%)	0.034*
ILD	7 (43.8%)	70 (80.5%)	7 (29.2%)	135 (57.0%)	<0.001*
**Laboratory Parameters**					
ESR (mm/1H, mean±SD)	29.3±17.1	34.1±20.3	29.8±23.4	34.5±26.7	0.456
CRP (mg/L, median[IQR])	5.8[1.3-10.0]	5.3[1.3-20.5]	9.0[1.2-40.6]	5.6[1.2-17.5]	0.345

Variable	ASyS-MAL (n=16)	ASyS-non-MAL (n=87)	non-ASyS-MAL (n=24)	non-ASyS-non-MAL (n=237)	*P*-value
CK (U/L, median[IQR])	978[364-3640]	288[84-841]	336[99-768]	314[73-1416]	0.012*
**Autoantibodies**					
ANA positive	6 (37.5%)	49 (56.3%)	15 (63.2%)	78 (56.9%)	0.345
MSAs positive	16 (100.0%)	87 (100.0%)	0 (0.0%)	0 (0.0%)	<0.001*
MAAs positive	10 (62.5%)	69 (79.3%)	11 (47.4%)	72 (52.3%)	<0.001*
Anti-Ro52 positive	10 (62.5%)	69 (79.3%)	11 (47.4%)	72 (52.3%)	<0.001*

Abbreviations: IIM, idiopathic inflammatory myopathy; ASyS, anti-synthetase syndrome; MAL, malignancy; IQR, interquartile range; ILD, interstitial lung disease; ESR, erythrocyte sedimentation rate; CRP, C-reactive protein; CK, creatine kinase; LDH, lactate dehydrogenase; ANA, antinuclear antibody; MSAs, myositis-specific antibodies; MAAs, myositis-associated antibodies.*$P < 0.05$.
