# Supplementary material for: Malignancy in anti-synthetase syndrome: clinical features and prognostic impact from a multicenter retrospective study
Source: Front Med (Lausanne). 2026 Mar 12;13:1780337. doi: 10.3389/fmed.2026.1780337 (PMC13018139; doi:10.3389/fmed.2026.1780337)
Supplement: Supplementary file 5 [file Table_4.pdf]

Supplementary Table S4. Subgroup Analysis: Comparison of ASyS Patients vs. Dermatomyositis (DM) Patients Only

Characteristic	ASyS Group (n=103)	DM Subgroup (n=195)	*P*-value
**Demographics**			
Age at onset (years), median (IQR)	55.0 (46.0–65.0)	52.0 (43.0–64.0)	0.223
Male sex, n (%)	31 (30.1%)	58 (29.7%)	0.950
**Clinical Features**			
Myositis, n (%)	76 (73.8%)	155 (79.5%)	0.348
ILD, n (%)	77 (74.8%)	142 (72.8%)	0.719
Heliotrope rash, n (%)	19 (18.4%)	135 (69.2%)	**<0.001**
**Malignancy Prevalence**			
Malignancy within 36-month window, n (%)	16 (15.5%)	19 (9.7%)	0.143
**Risk Analysis for Malignancy**			
Unadjusted OR (95% CI)	1.70 (0.84–3.45)	Reference	0.142
Adjusted OR* (95% CI)	2.41 (1.05–5.55)	Reference	**0.039**

*Adjusted for age and sex.

Interpretation: When compared specifically to the DM subgroup (rather than the heterogeneous non-ASyS group), ASyS remained significantly associated with increased malignancy risk after adjustment for age and sex (aOR 2.41, 95% CI 1.05–5.55, P=0.039). This sensitivity analysis addresses concerns about heterogeneity in the non-ASyS comparator group.
